# Influence of Incubation Temperature on 9,10-Anthraquinone-2-Sulfonate (AQS)-Mediated Extracellular Electron Transfer

**DOI:** 10.3389/fmicb.2019.00464

**Published:** 2019-03-06

**Authors:** Wei Liu, Yundang Wu, Tongxu Liu, Fangbai Li, Hui Dong, Meiqing Jing

**Affiliations:** ^1^College of Materials and Energy, South China Agricultural University, Guangzhou, China; ^2^Guangdong Key Laboratory of Integrated Agro-Environmental Pollution Control and Management, Guangdong Institute of Eco-Environmental Science and Technology, Guangzhou, China

**Keywords:** extracellular electron transfer, temperature, redox transformation, mediator, biofilm

## Abstract

The electron shuttling process has been recognized as an important microbial respiration process. Because the incubation temperature can influence both the reactivity of electron mediators and cell growth, it may also affect the electron-shuttle-mediated extracellular electron transfer (EET) process. Here, the effect of incubation temperature (22–38°C) was investigated in a bioelectrochemical system (BES) using *Shewanella oneidensis* MR-1 and 50 μM of 9,10-anthraquinone-2-sulfonate (AQS). We found that current generation increased as the temperature was increased from 22 to 34°C and then decreased sharply at 38°C. The biofilm biomass, as indicated by the total protein extracted from the electrode, increased as the temperature increased from 22 to 34°C and then decreased at 38°C, mirroring the current generation results. These results were further confirmed by increasing the temperature slowly, step-by-step, in a single BES with a constant biofilm biomass, suggesting that the EET rates could be substantially influenced by temperature, even with the same biofilm. The effects of temperature on the AQS bioreduction rate, *c*-type cytochrome (*c*-Cyts)-bound-cofactor-mediated EET, the AQS mid-point potential, and the AQS diffusion coefficient were studied. From these results, we were able to conclude that temperature influenced the EET rates by changing the *c*-Cyts-bound-cofactor-mediated EET process and the AQS bioreduction rate, and that the change in biofilm formation was a dominant factor influencing the overall EET rates. These findings should contribute to the fundamental understanding of EET processes. Moreover, optimization of the operating parameters for current generation will be helpful for the practical application of bioelectrochemical techniques.

## Introduction

Extracellular electron transfer (EET) is a key process of extracellular respiration, which is an important part of microbial anaerobic metabolism (Lovley et al., 1987; Shi et al., 2007, 2009, 2016; Calandra et al., 2016; Light et al., 2018). As this process is active in the epigeosphere, it has an impact on the fate of trace metals and nutrients and on the degradation of organic matter (Ohtsuka et al., 2013; Zhou et al., 2016; Han et al., 2018). Owing to the EET ability of microbes, microbial fuel cells can be constructed to generate bioelectricity (Logan, 2009). Because some organic materials, such as quinone compounds, can act as electron shuttles to facilitate electron transfer in bioelectrochemical systems (BESs) (Klüpfel et al., 2014; Yuan et al., 2017), extensive attention has been paid to quinone-compound-mediated EET processes (Watanabe et al., 2009; Niedźwiecka et al., 2017; Wu et al., 2018; Yamamura et al., 2018).

Quinone-compound-mediated EET includes two steps, namely, bioreduction of quinone to hydroquinone and chemical oxidation of hydroquinone to quinone (Wu et al., 2014). The dynamics of the redox transformations of quinone compounds may directly affect EET rates (Li et al., 2013, 2014), thus indirectly affecting cell growth, by changing the metabolic rates of carbon sources (Gralnick and Newman, 2007). Hence, the biofilm in the BES, which determines current generation, may also be influenced by the dynamics of the quinone redox transformations. In addition, the understanding of the mechanism of *c*-type cytochrome (*c*-Cyts)-bound-cofactor-mediated EET has become better in recent years. It has been reported that flavins secreted by *Shewanella oneidensis* MR-1 primarily act as *c*-Cyts-bound cofactors the accelerate EET, rather than as free soluble shuttles (Okamoto et al., 2013; Xu et al., 2016). Hence, the effects of environmental factors on *c*-Cyts-bound-cofactor-mediated EET also need to be properly understood.

Temperature is an important environmental factor that affects microbial activity, and its effects on microbial growth have been extensively reported. For example, it has been shown that *S. oneidensis* MR-1 can survive in a wide temperature range (3–35°C) (Abboud et al., 2005), that the metabolism of *Shewanella* (i.e., fatty acid biosynthesis) changes with temperature (Wang et al., 2009), and that its iron bioreduction rate decreases when the temperature decreases from 37 to 4°C (Picard et al., 2014). However, few previous studies have considered the impact of temperature on the specifics of quinone-compound-mediated EET. It has been recognized that changes in environmental factors, such as pH, significantly impact biofilm growth and the quinone redox potential (Wu et al., 2016), resulting in large changes in the produced current. Nevertheless, it remains unclear whether, or how, transient or longer-term temperature differences affect the formation and redox properties of the biofilm, which could in turn influence the quinone-compound-mediated EET process.

Thus, to investigate these issues in detail, a BES was constructed with *S. oneidensis* MR-1 as a model strain. A quinone compound (9,10-anthraquinone-2-sulfonate, AQS), which has a similar structure to anthraquinone-2,6-disulfonate (AQDS), was chosen as the model mediator because our previous studies (Li et al., 2013, 2014; Wu et al., 2014) demonstrated that the enhancing effects of AQS on EET were higher than those of AQDS. The objectives of this study were to (1) clarify how transient temperature changes and long-term temperature differences affect current generation in a BES with AQS; (2) quantitatively investigate the factors that influence the AQS-mediated EET process; and (3) determine the underlying mechanism responsible for the temperature effect on AQS-mediated EET.

## Materials and Methods

### Materials and BES Setup

*Shewanella oneidensis* MR-1, purchased from the Marine Culture Collection of China (China), was aerobically incubated in lysogeny broth (LB) medium, at 30°C while being continuously shaken at 180 rpm. When the cell suspension was in the logarithmic phase, it was centrifuged, washed, and then diluted to the target concentration for the following experiments. Each BES, equipped with a carbon cloth (2 cm × 2 cm) working electrode, titanium counter electrode, and calomel reference electrode, was incubated anaerobically at a constant potential of 441 mV vs. Standard Hydrogen Electrode (SHE). Phosphate was used as the pH buffer (pH = 7.0). A solution of AQS (AR, 98.0%) was obtained from Acros (China). All other chemicals were purchased from the Guangzhou Chemical Reagent Factory (China).

### Spectral Measurements

By using UV-visible diffuse-transmittance absorption spectroscopy, AH_2_QS can be directly detected in living cell suspensions. A sealed cuvette was used as the reactor, to which AQS (50 μM), MR-1 (OD_600_ = 1.0), and lactate (50 mM) were added. The spectra of AH_2_QS were recorded *in situ* using a diffuse-transmittance spectrophotometer (UV-2600, Shimadzu) equipped with an integrating sphere. A standard solution of AH_2_QS was prepared in an anaerobic chamber, with sodium hydrosulfite used to reduce AQS to AH_2_QS. To calibrate the AH_2_QS concentration, AH_2_QS spectra (0–50 μM), with excess sodium hyposulfite as reducer, were collected, and each AH_2_QS concentration was determined from the absorbance peak at 382 nm. The standard AH_2_QS curve had a slope of 153.4 (*R*^2^ = 0.9996). For the treatment with cell suspensions and AQS, the AH_2_QS concentration was determined according to the difference between the spectrum of MR-1 with AH_2_QS and that of MR-1 only.

### Electrochemical Measurements

An electrochemical workstation (Autolab PGSTAT 302N, Metrohm, Switzerland) was used for electrochemical impedance spectroscopy (EIS) measurements. EIS spectra of the BESs were obtained by applying sinusoidal perturbations of ±10 mV over the open circuit voltage at frequencies from 10^−2^ to 10^5^ Hz. Cyclic voltammetry (CV) and differential pulse voltammetry (DPV) tests were conducted using a potentiostat (CHI660D, Chenhua Co., Ltd., China). CV of AQS was performed at various scanning rates (50–400 mV s^−1^). Carbon cloth (2 cm × 2 cm) electrodes were used as working electrodes in the electrochemical systems.

### Biofilm Characterization

The morphologies of the biofilms on the electrodes were characterized using scanning electron microcopy (SEM; S-3000N, Hitachi, Japan) and fluorescence microscopy (Axio Scope A1, Carl Zeiss, Germany). The carbon cloth electrodes with biofilms were removed after incubation for 3 days, gently washed in water, and then treated with glutaraldehyde (2.5%), osmic acid, and ethanol. After freeze-drying and coating with evaporated platinum, the washed biofilm samples were imaged using SEM. The cells were then dyed with 4′,6-diamidino-2-phenylindole before fluorescence microscopy imaging.

The biofilm was then quantified by extracting the total biofilm protein with a boiling 0.2 M NaOH solution (Zhao et al., 2013). A carbon cloth sample (2 cm × 0.66 cm) was placed into a 2 mL sealed tube, together with 0.5 mL NaOH and some glass beads, and the tube was then subjected to a super high-speed vortex (6.5 m s^−1^, 45 s) using a homogenizer (FastPrep-24, MP, United States). The mixture was then centrifuged at 8000 *g* for 3 min, and finally, the supernatant was collected for protein quantification. The supernatant was then quantified by Coomassie blue staining via a protein quantification kit (C503041-1000 Modified Bradford Protein Assay Kit, Sangon Biotech, China).

## Results

### Electricity Generation at Different Temperatures

**FIGURE 1:**
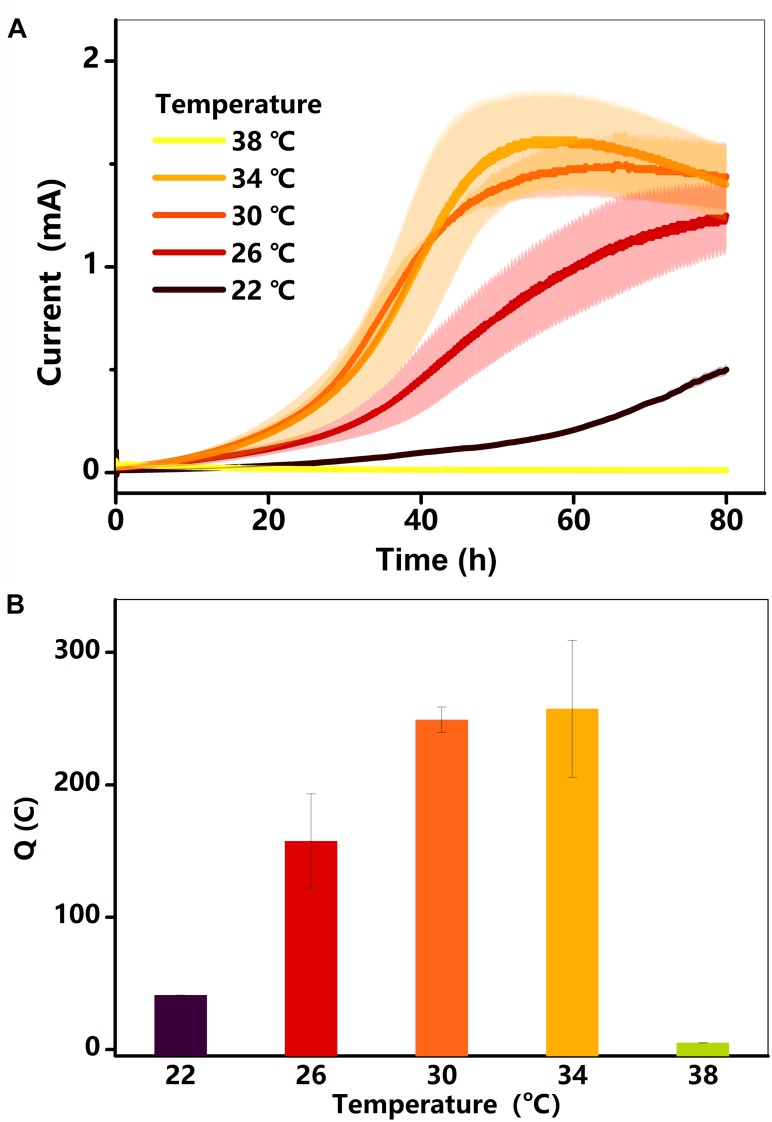
**(A)** Current (*I*, mA) in BESs against time at different temperatures (22–38°C); **(B)** total charge as a function of temperature. Testing conditions: 50 μM AQS, 50 mM lactate, 200 mM phosphate as pH buffer (pH = 7.0), OD_600_ = 1.0, and external potential of 421 mV vs. SHE.

Electricity generation in the BES was examined at different temperatures. As shown in Figure 1A, the current in each treatment (22–34°C) started increasing after a hysteresis period of approximately 5 h, reaching a maximum value within 72 h. Electricity generation was extremely low at 38°C, lasting for 40 h before decreasing to near zero. The total charge (*Q*) for each treatment is shown in Figure 1B. The *Q*, which was only 41°C at 22°C, increased gradually to 257°C at 34°C, and then decreased substantially to 5°C at 38°C. As the current and charge changed, the rate of lactate consumption must have changed (Pinchuk et al., 2011; Saito et al., 2016).

Because the concentration of lactate can influence the physiology of *S. oneidensis* MR-1, the effect of lactate concentration (10, 20, 30, 40, and 50 mM) on current generation was examined in the BES at 34°C. The results (Supplementary Figure S1) suggested that the generated current was very similar for all lactate concentrations between 10 and 50 mM. It has previously been reported that the current generated without AQS is much lower than that generated with AQS (Wu et al., 2016). The treatments with and without exogenous AQS are very different. The main purpose of this study is to investigate the influence of temperature on the AQS-mediated EET process quantitatively.

### Biofilm Formation at Different Temperatures

**FIGURE 2:**
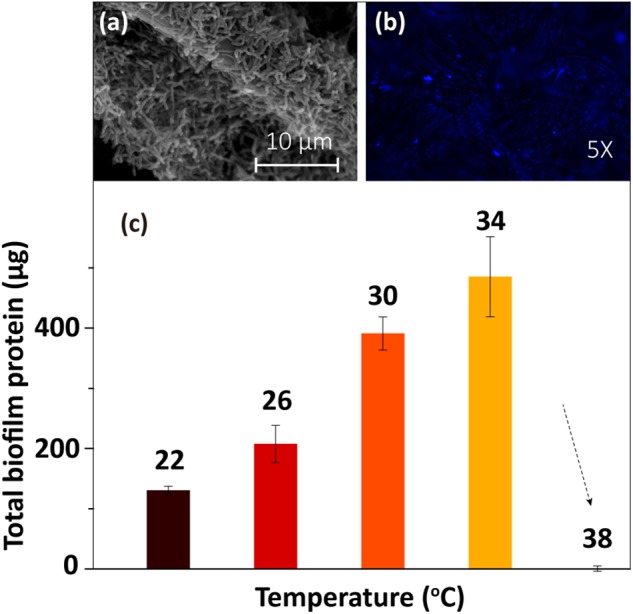
Biofilm morphology at 30°C: **(a)** SEM image and **(b)** fluorescence microscopy image. **(c)** Total biofilm protein at different temperatures.

The SEM and fluorescence microscopy images of the biofilm sample at 30°C (Figure 2a,b) showed that the electrode surface was fully covered with biofilm cells. To determine the amount of biofilm formed under different temperatures, the total protein on the electrode was extracted and then quantified using Coomassie blue staining via a protein quantification kit. The results (Figure 2c) showed that the total biofilm protein amount on the electrode increased gradually as the temperature rose from 22 to 34°C and then sharply decreased as the temperature increased further (from 34 to 38°C). The low concentration of protein at 38°C implied that the cells were dead at this temperature. Thus, this temperature is not suitable for the anaerobic survival of MR-1 cells (Abboud et al., 2005).

### Electricity Generation at Different Temperatures With a Constant Biofilm

**FIGURE 3:**
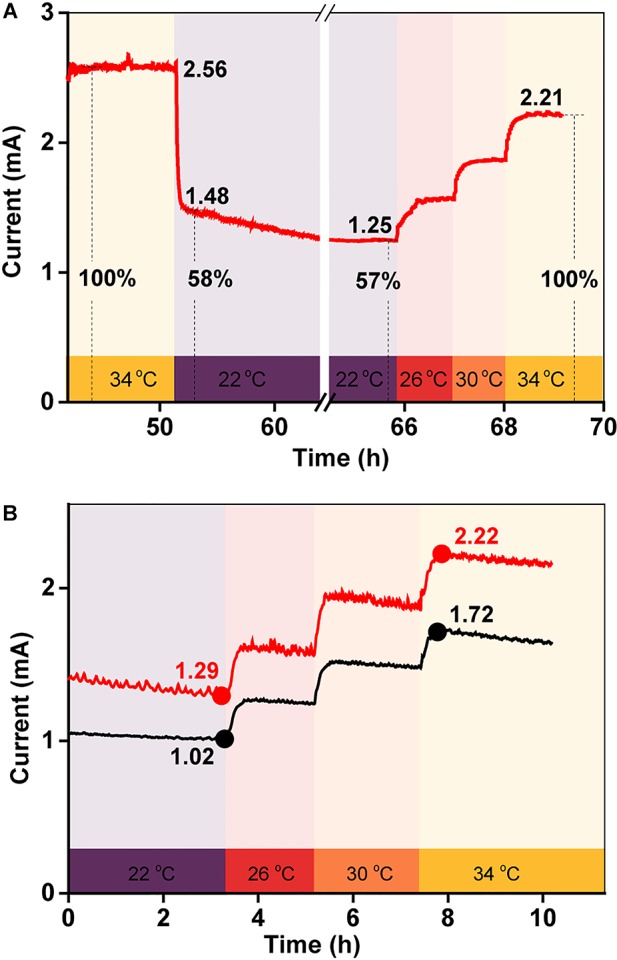
**(A)** Current generation in a BES when the temperature was quickly decreased from 34 to 22°C, and then gradually increased back to 34°C over 3 h; **(B)** replicates of **(A)**.

To examine the effect of temperature on electricity generation with the same biofilm, current generation was examined in a BES as the temperature was quickly reduced from 34 to 22°C and then gradually increased back to 34°C over a period of 3 h. As shown in Figure 3A, the current decreased sharply after the initial temperature decrease, suggesting that the BES electron transfer ability was very sensitive to temperature. Then, as the temperature was gradually restored back to 34°C, the current increased, step-by-step, with the increasing temperature. The percentage by which the current intensity decreased was consistent with the increased current intensity, which suggested that the temperature-dependent change in the current output was reversible. To confirm this phenomenon, this test was repeated twice. The results in Figure 3B show that the percentage by which the current increased with the step-by-step temperature increase was similar for each.

### Quantitative Analysis of the Effect of Temperature on Current Generation

To quantify the influence of each factor on total current production, the maximum current at 34°C was defined as 100%, meaning that the current decreased by 58% when the temperature was rapidly reduced from 34 to 22°C (Figure 3A,B). As the biofilm biomass remained constant during this period, the decrease in current generation was mainly caused by a change in the redox reactivity of AQS and the metabolic activity of the cells. However, after long-term incubation, the maximum current intensity decreased to 31% when the temperature decreased to 22°C (Figure 1A). The biomass changed substantially during this period, indicating that the extra current decrease was caused by the decrease in biofilm biomass.

### Electrochemical Properties of the Biofilm at Different Temperatures

**FIGURE 4:**
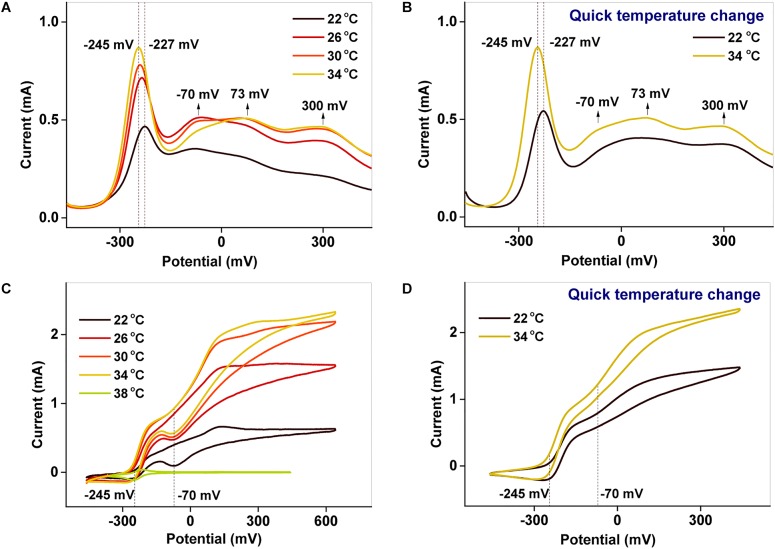
**(A,B)** DPV and **(C,D)** CV characterizations of biofilms cultured at different temperatures. In **(B,D)**, the temperature was suddenly lowered from 34 to 22°C.

To examine the redox properties of the biofilms, electrochemical characterizations were performed after potentiostatic incubation. The DPV results (Figure 4A,B) allow various processes occurring in the biofilm to be distinguished. The peaks around −227 to −245 mV and −70 mV correspond to free AQS and *c*-Cyts-bound cofactor, respectively (Wu et al., 2016; Xu et al., 2016), whereas the peaks at 73 and 300 mV reflect direct *c*-Cyts redox reactions (Okamoto et al., 2013, 2014b; Xu et al., 2016). When the incubation temperature was reduced from 34 to 22°C, the peak potential of free AQS increased slightly from −245 to −227 mV, and the peak shapes of the *c*-Cyts-bound cofactor (−70 mV) and free *c*-Cyt (73 and 300 mV) showed obvious changes (Figure 4A). However, the shapes of these peaks remained stable when the temperature decreased from 34 to 22°C instantaneously (Figure 4B).

Using CV analysis, the catalytic current generated by each process could be studied and the contribution from each process to the catalytic current could be distinguished. From the CV results in Figure 4C,D, two significant current increases were observed at −245 and −70 mV, representing the free AQS mediation process and the *c*-Cyt-bound cofactor associated transport, respectively. Meanwhile, free *c*-Cyt (+73 mV) exhibited a very weak contribution to the catalytic current. As shown in Figure 4C, as the temperature was decreased, the peak at −70 mV decreased significantly, whereas the peak at −245 mV decreased only slightly from 34 to 26°C before decreasing much more as the temperature decreased further (from 26 to 22°C). As shown in Figure 4D, the peak corresponding to the mediation process only decreased slightly when the temperature was suddenly lowered from 34 to 22°C, whereas the peak corresponding to *c*-Cyts-bound cofactor associated transport decreased significantly. This phenomenon was verified in triplicate. These results indicated that both the mediation process and *c*-Cyts-bound cofactor associated electron transfer were changed by temperature after long-term incubation, whereas only *c*-Cyts-bound cofactor associated electron transfer was changed significantly when the temperature decreased quickly from 34 to 22°C.

**FIGURE 5:**
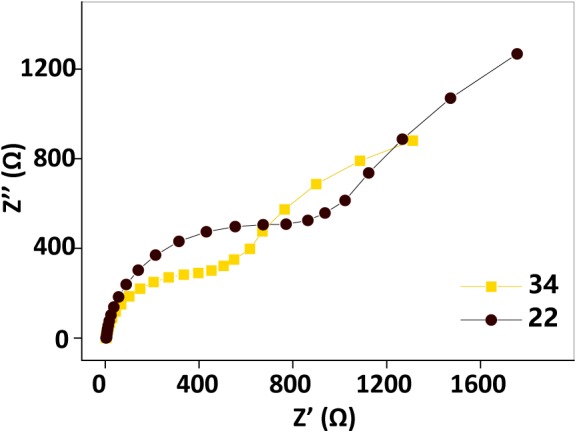
Electrochemical impedance spectroscopy spectra before (34°C, orange squares) and after (22°C, black dots) the temperature changing operation. Test conditions: 50 μM AQS, 50 mM lactate, 200 mM phosphate as pH buffer (pH = 7.0), OD_600_ = 1.0, and external potential of 421 mV vs. SHE.

Electrochemical impedance spectroscopy tests performed before and after the temperature changing operation (Figure 5) showed that the impedance also increased as the temperature decreased. This observation also implied that factors other than the biofilm biomass, such as the AQS redox properties, the properties of the *c*-Cyt-bound cofactor, and the metabolic activity of the microbes, may also be temperature dependent, causing current generation in the BES with a fixed biofilm to change with temperature.

### Redox Transformation of AQS at Different Temperatures

It has been documented that electron transport during AQS-mediated EET processes occurs via the redox cycling of AQS, including the reduction of AQS to AH_2_QS by MR-1 and the electrochemical oxidation of AH_2_QS to AQS on the surface of the electrode. The *in situ* kinetics of quinone compound reduction can be studied in living bacterial suspensions using diffuse-transmittance spectroscopy (Nakamura et al., 2009; Han et al., 2016; Liu et al., 2017). Therefore, the bioreduction of AQS and the electrochemical oxidation of AH_2_QS were investigated at different temperatures.

**FIGURE 6:**
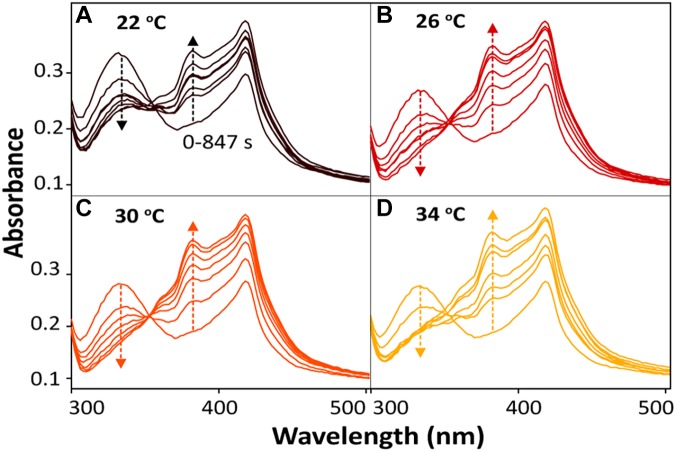
*In situ* spectral kinetics of AQS reduction at different temperatures: **(A)** 22°C, **(B)** 26°C, **(C)** 30°C, and **(D)** 34°C. The concentrations of AH_2_QS were calculated from the absorbance peak at 382 nm. Test conditions: 50 μM AQS with MR-1 (OD_600_ = 1.0) and 50 mM lactate.

**FIGURE 7:**
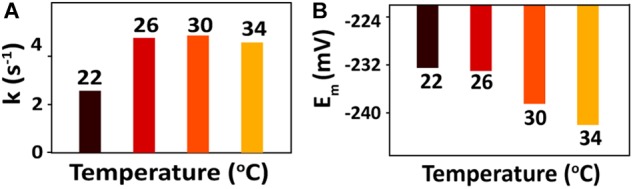
**(A)** Calculated rate constants for AQS bioreduction at different temperatures. **(B)** CV midpoint potentials (*E*_m_) of AQS at different temperatures.

For the bioreduction of AQS to AH_2_QS without an electrode, similar patterns were observed for the spectral kinetics at different temperatures de (Figure 6A–D). The peaks at 330 and 382 nm were attributed to AQS and AH_2_QS, respectively. To demonstrate the kinetics of AQS reduction to AH_2_QS clearly, the concentrations of AH_2_QS at different temperatures were plotted as a function of time. As the concentration of lactate (50 mM) in the system was much higher than that AQS (50 μM), the reduction of AQS can be considered a pseudo-first-order reaction. As shown in Figure 7A, the rate constants increased at temperatures of 22–26°C and then slightly decreased at higher temperatures (30–34°C).

**FIGURE 8:**
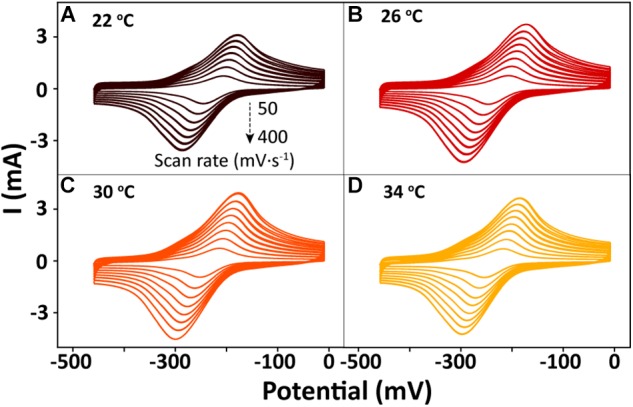
Cyclic voltammetry results for AQS at different scan rates (50–400 mV s^−1^) and temperatures: **(A)** 22°C, **(B)** 26°C, **(C)** 30°C, and **(D)** 34°C.

After bioreduction of AQS to AH_2_QS, electrochemical oxidation of AH_2_QS will ultimately result in current generation. To examine the differences in the AQS redox reactions on the electrode surface at different temperatures, CV of AQS was conducted at various scan rates (50–400 mV s^−1^). As shown in Figure 8A–D, an increase in the current as the scan rate increased was accompanied by, a positive shift of the oxidation peak and a negative shift of the reduction peak. The midpoint potential (Figure 7B) decreased slightly, from −232.5 to −242 mV, with increased temperature (22–34°C).

## Discussion

### Role of Temperature-Dependent Biofilm Formation

The biofilm, as the driving force for EET, has been observed to be affected by the incubation temperature (Tango et al., 2018; Zhang et al., 2018), and the changes in biofilm biomass may significantly influence the EET process. To examine the effect of temperature-dependent biofilm formation on electricity generation, the linear regression of total charge (*Q*) as a function of total biofilm protein was analyzed. A good linear relationship (*R*^2^ = 0.91) was observed between the *Q* values and total biofilm protein, which implied that biofilm cell growth is an essential factor that affects EET at different temperatures. The amount of total biofilm protein increased as the temperature rose from 22 to 34°C, indicating a corresponding increase in the lactate consumption rate. As shown by Rxn. 1, high lactate consumption rates could increase the amount of available electrons, which would be favorable for AQS reduction (Rxn. 2 and Figure 8) and current generation (Rxns. 3 and 4). In addition, if the maximum current at 34°C was defined as 100% as shown in Supplementary Figure S3a, the maximum current decreased to 31% when the biofilm was incubated at a temperature of 22°C (Supplementary Figure S3c). This current intensity was almost two times lower than that obtained when the temperature was changed instantaneously (Supplementary Figure S3b). This difference in behavior might be caused by changes in the biofilm biomass in the former system. Notably, the current also decreased when the temperature was reduced from 34 to 22°C in a single BES with the same biofilm (Supplementary Figure S3b). Therefore, factors other than the biofilm must contribute to the changes in current generation at different temperatures.

### Free AQS-Mediated EET Processes in BESs

It is known that the reduction of an electron acceptor (i.e., AQS) can be coupled to an oxidizing electron donor (lactate) during current generation by MR-1 (Coursolle and Gralnick, 2010; Pinchuk et al., 2011), with the diffusion of reduced AQS to the anode electrode resulting in current generation. The potential losses can drive electron flow from the electron donor to the anode. For lactate oxidation, the following half-cell reaction (Rxn. 1) determines the electron production rates and the concomitant anode potential.

$${\text{C}}_{3}{\text{H}}_{5}{\text{O}}_{3}^{-}+2{\text{H}}_{2}\text{O}\to {\text{C}}_{2}{\text{H}}_{3}{\text{O}}_{2}^{-}+{\text{HCO}}_{3}^{-}+5{\text{H}}^{+}+4{\text{e}}^{-}\;(\mathrm{Rxn}.1)$$

According to the Nernst equation, the specific theoretical redox potential of the electron donor can be determined using Eq. 1.

(1) $$E_{{\text{C}}_{3}{\text{H}}_{5}{\text{O}}_{3}^{-}}=E_{{\text{C}}_{3}{\text{H}}_{5}{\text{O}}_{3}^{-}}^{0}-\frac{RT}{4F}\ln\frac{[{\text{C}}_{2}{\text{H}}_{3}{\text{O}}_{2}^{-}][{\text{HCO}}_{3}^{-}]{\left[{\text{H}}^{+}\right]}^{5}}{\left[{\text{C}}_{2}{\text{H}}_{3}{\text{O}}_{3}^{-}\right]}$$

As a result of the oxidation of lactate, the electrons generated can be captured by AQS to produce AH_2_QS (Rxn. 2).

$$\text{AQS}+2{\text{H}}^{+}+2{\text{e}}^{-}↔{\text{AH}}_{2}\text{QS }(\mathrm{Rxn}.2)$$

The redox potential of AQS (*E*_AQS_) can be calculated using Eq. 2.

(2) $$E_{\text{AQS}}=E_{\text{AQS}}^{0}-\frac{RT}{2F}\ln\frac{\left[{\text{AH}}_{2}\text{QS}\right]}{[\text{AQS}]{\left[{\text{H}}^{+}\right]}^{2}}$$

Hence, at a fixed pH, the driving force for lactate oxidation and AQS reduction ${(}_{\Delta E=E_{{\text{C}}_{3}{\text{H}}_{5}{\text{O}}_{3}^{-}}-E_{\text{AQS}}})$ can be influenced by both the temperature and the concentrations of lactate and AQS, which is why the peak potentials of AQS changed, as shown in Figure 4A,B, 7B.

However, as a metabolic process determined by microbial activity, the oxidation of lactate by MR-1, as well as the biofilm biomass, was sensitive to temperature. The peak of the free AQS-mediated process at −245 mV exhibited a noticeable decrease when the temperature decreased gradually from 34 to 22°C (Figure 4C), but this only showed a slight decrease when the temperature was decreased rapidly (Figure 4D). This difference suggested that the change in the AQS bioreduction rate likely has an important effect on current generation in the long-term incubation system. The change in the AQS bioreduction rate constant (Figure 7A) showed a similar tendency as the change of the peak in Figure 4C, which confirmed the importance of the AQS bioreduction process.

After AQS was reduced to AH_2_QS, it then diffused to the electrode surface and concomitantly transferred electrons to the anode, resulting in the generation of current (Brutinel and Gralnick, 2012).

$${\text{AH}}_{2}{\text{QS}}_{\text{red}}+\text{Anode}\to \text{Anode}\cdot {\text{AH}}_{2}\text{QS }(\mathrm{Rxn}.3)$$

$$\text{Anode}\cdot {\text{AH}}_{2}\text{QS}\to \text{AQS}+{\text{e}}^{-}\;(\mathrm{Current})\;(\mathrm{Rxn}.3)$$

The peak current (Figure 8A–D) exhibited a linear relationship with the square root of the scan rate (*v*^1/2^) (Supplementary Figure S2), indicating that a diffusion process (Rxn. 3) was the rate-determining step in the electrochemical oxidation of AH_2_QS on the electrode (Richter et al., 2009).

The transport of soluble AH_2_QS to the anode (Rxn. 3) is a diffusion process governed by Fick’s law (Torres et al., 2009). As the diffusion coefficient at different temperatures follows the Einstein relation (*D* = *KT*/6πη*r*), Fick’s law can be written as shown in Eq. 3.

(3) $$j=\mathrm{nF}\left(\frac{\mathrm{KT}\Delta [{\text{AH}}_{2}\text{QS}]}{6\pi \eta r\Delta z}\right)$$

where *j* is the current density (A m^−2^), *nF* is a conversion factor from moles to coulombs, *K* is Boltzmann’s constant, *T* is the absolute temperature, Δ[AH_2_QS] is the concentration gradient of AH_2_QS (mol m^−3^), π is the circumference ratio, η is the coefficient of viscosity of the solution (Pa s), *r* is the hydrodynamic radius (m), and Δ*z* is the transport distance (m). Hence, it is clear that the current density (*j*) can be positively affected by temperature. However, based on the Einstein relation, a 1.05-fold change in the value of *D* should be observed between 22 and 38°C (295 K/311 K). Notably, we observed a larger difference in our current production values, indicating that the changes in the diffusion process probably have a very limited influence on current generation.

### Cytochrome-Bound-Co-factor-Mediated EET in BESs

In addition to the free AQS-mediated process, the *c*-Cyts-bound cofactor can also be involved in electron transfer. As reported previously, low concentrations of flavin can be bound to *c*-Cyts, and as the *c*-Cyts-bound cofactor is obviously different from free flavin, it can be used to regulate the extent of EET processes (Okamoto et al., 2013, 2014a,c). Hence, the roles of temperature-dependent, *c*-Cyts-bound-cofactor-mediated electron transfer processes are considered further below.

Figure 4A,B exhibit peaks at −245 and −70 mV corresponding to free AQS and the *c*-Cyts-bound cofactor, respectively. Thus, the catalytic currents generated at −245 and −70 mV, as depicted in Figure 4C,D, were contributed from the free AQS-mediated process and the *c*-Cyts-bound-cofactor-mediated process, respectively. In the long-term incubation experiments, the catalytic current from both the free AQS-mediated process and the *c*-Cyts-bound-cofactor-mediated process decreased as the temperature decreased from 34 to 22°C (Figure 4C). However, in the instantaneous temperature change experiments, the catalytic current from the *c*-Cyts-bound-cofactor-mediated EET process decreased significantly, whereas that from the free AQS-mediated process only decreased slightly (Figure 4D). These results suggested that the current variation in the instantaneous temperature change experiment was mainly caused by a decline in *c*-Cyts-bound-cofactor-mediated EET, whereas both weaker AQS-mediated and *c*-Cyts-bound-cofactor-mediated EET contributed to the total current decrease during the long-term incubation experiment.

The bioreduction rate of AQS is mainly limited by the metabolic rate of microbes. As changes in the rate of microbial metabolism probably require a certain period of time, the AQS reduction rate did not change much in the short-term incubation and instantaneous temperature change experiments. Hence, as shown in Figure 7A, the rate constants of AQS reduction only changed slightly. However, flavin as a bound cofactor for accelerating EET is involved in a biochemical process on the outer surface of the cell. It has been reported that this process can be regulated by the properties of *c*-Cyts, and *S. oneidensis* MR-1 has the capacity to use flavin as a regulator to control the extent of EET processes (Okamoto et al., 2013). Hence, the response of this biochemical process to changes in temperature may be faster than that of microbial metabolism, resulting in the *c*-Cyts-bound-cofactor-mediated EET process being more susceptible to sudden temperature changes.

Although MR-1 has been shown to grow slower at 34°C than at 30°C, the reported optimum temperature for manganese reduction by MR-1 is 35°C (Myers and Nealson, 1988). Hence, the optimum temperature of MR-1 in different system can be different. In this study, 34°C was the optimum temperature, probably because in the presence of exogenous AQS, the change in temperature not only changes the physiology of MR-1 but also changes the electron shuttling properties of AQS. Specifically, it can influence the AQS midpoint potential, the AQS bioreduction process, the AQS diffusion coefficient, and *c*-Cyts-bound-cofactor-mediated EET. These factors might contribute to the observation of a lower optimum temperature of 34°C in this study.

Here, the EET capacity increased as the temperature rose from 22 to 34°C and then decreased sharply at 38°C. Besides the cell death observed at 38°C, five key factors, namely, the biofilm biomass, *c*-Cyts-bound-cofactor-mediated EET, the AQS bioreduction rate, the AQS midpoint potential, and the AQS diffusion coefficient, could be affected by changing the incubation temperature. The temperature-dependent AQS midpoint potential and the AQS diffusion coefficient contributed little to the variations in total electricity generation at different temperatures, whereas the temperature-dependent biofilm biomass, *c*-Cyts-bound-cofactor-mediated EET, and the AQS bioreduction rate dominated the observed electricity generation variation. As the ambient temperature changes often in natural environments, it is an important environmental factor that influences natural microbial processes such as biofilm formation and microbe-mineral electron transfer. Thus, our findings provide an improved fundamental understanding of EET processes and will aid in the practical application of bioenergy techniques via optimization of the operating parameters for current generation in relevant BESs such as microbial fuel cells.

## Author Contributions

WL, YW, FL, and TL conceived and designed the experiments. WL, YW, and TL were responsible for drafting the article. WL, YW, HD, and MJ were involved in the experiments preformation and data analysis. All authors approved the final version of the manuscript.

## Conflict of Interest Statement

The authors declare that the research was conducted in the absence of any commercial or financial relationships that could be construed as a potential conflict of interest.

## Supplementary Material

FIGURE S1 The effect of lactate concentration (10, 20, 30, 40, and 50 mM) on current generation.

FIGURE S2 The peak current in Figure 8A–D versus the square root of the scan rate.

FIGURE S3 The normalized current output at different conditions: **(a)** Maximum current when the biofilm was incubated at a temperature of 34°C. It was defined as 100%. **(b)** Current after the temperature was changed instantaneously from 34 to 22°C. **(c)** Maximum current when the biofilm was incubated at a temperature of 22°C.
