# Supplementary figures and images for: Influence of Incubation Temperature on 9,10-Anthraquinone-2-Sulfonate (AQS)-Mediated Extracellular Electron Transfer
Source: Front Microbiol. 2019 Mar 6;10:464. doi: 10.3389/fmicb.2019.00464 (PMC6414795; doi:10.3389/fmicb.2019.00464)

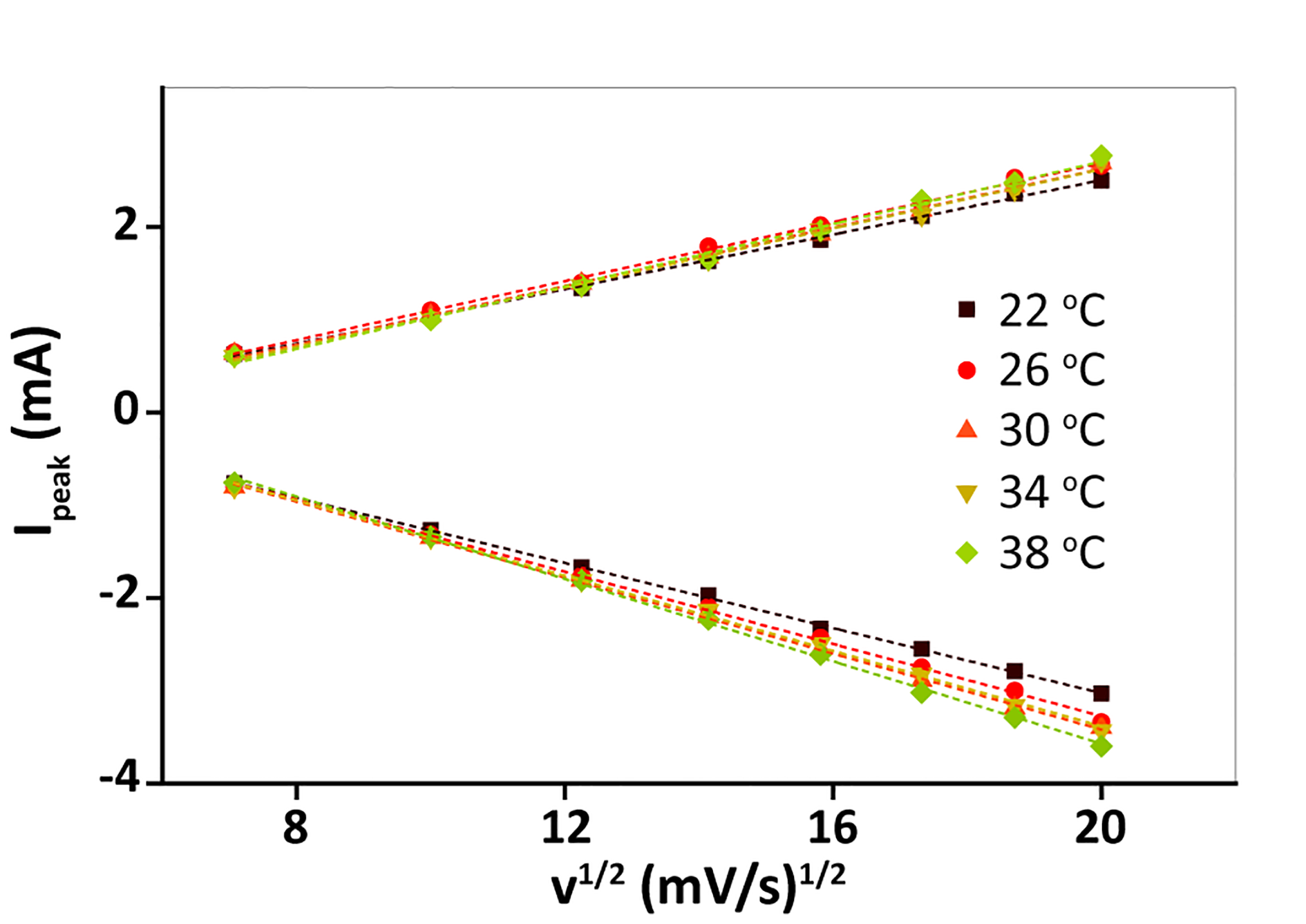

Supplement: FIGURE S2 — The peak current in Figure 8A–D versus the square root of the scan rate. [file Image_2.TIF]

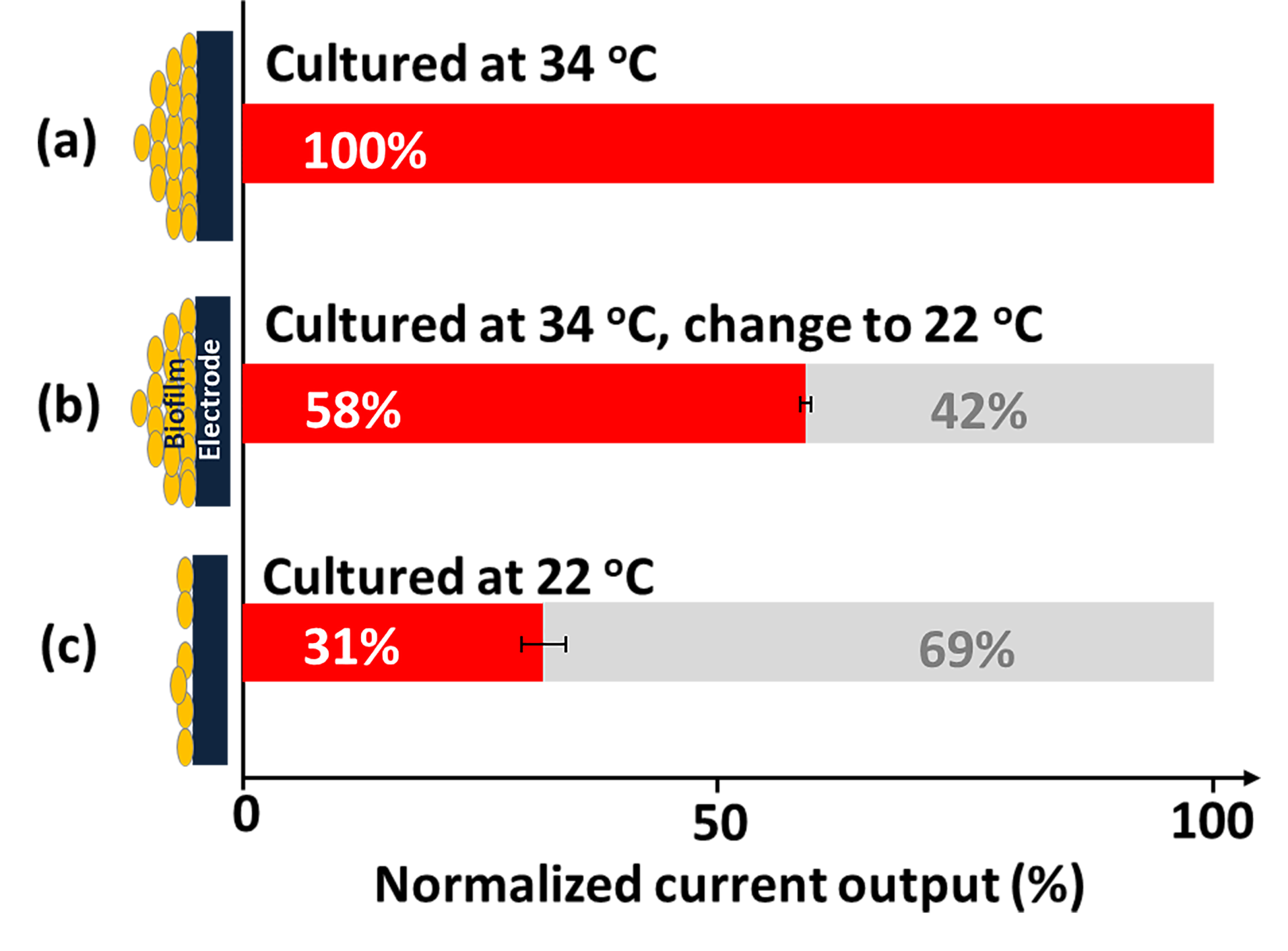

Supplement: FIGURE S3 — The normalized current output at different conditions: (a) Maximum current when the biofilm was incubated at a temperature of 34°C. It was defined as 100%. (b) Current after the temperature was changed instantaneously from 34 to 22°C. (c) Maximum current when the biofilm was incubated at a temperature of 22°C. [file Image_3.TIF]
